# The Treatment of Convulsions in Children

**Published:** 1891-05

**Authors:** 


### Selections and Abstracts

THE TREATMENT OF CONVULSIONS IN CHILDREN.—In a case of convulsions in a child, if the patient is cyanotic, a few whiffs of amyl nitrite, followed by inhalations of chloroform to relax spasm, should be given. These should be followed as soon as possible by hypodermic injection of tincture of veratrum viride, one-half drop for each year of age up to six years. The veratrum may be repeated in half an hour or an hour, if the convulsions recur. If the convulsions are uræmic, a small dose of morphine may be added or given separately.

In all the cases in which I have employed the foregoing treatment the effect was remarkably good, and in but one case have I had to repeat the injection of veratrum. The convulsions cease, the muscles relax, the pulse becomes slower, the temperature falls, and the skin becomes moist.

Indeed, the danger is over in less time than by any other means I have seen employed.

Appropriate after-treatment, as may be indicated, should of course be adopted.—*Lancet Clinic.*

Appomorphia, hypodermically, would meet the indications in a majority of cases of convulsions in children.

